# The most influential COVID-19 articles: A systematic review

**DOI:** 10.1016/j.nmni.2023.101094

**Published:** 2023-02-11

**Authors:** Suhaib JS. Ahmad, Konstantinos Degiannis, Joseph Borucki, Sjaak Pouwels, David Laith Rawaf, Marion Head, Chun Hei Li, Rami Archid, Ahmed R. Ahmed, Anil Lala, Wasif Raza, Katie Mellor, Doerte Wichmann, Aristomenis Exadaktylos

**Affiliations:** a Department of General Surgery, Betsi Cadwaladr University Health Board, Wales, UK; b Department of Trauma, Hand and Reconstructive Surgery, University Hospital of Saarland, University of Saarland, Homburg, Germany; c Norfolk and Norwich University Hospitals NHS Foundation Trust, Norwich, UK; d Department of General, Abdominal and Minimally Invasive Surgery, Helios Klinikum Krefeld, Germany; e WHO Collaborating Centre for Public Health Education & Training, Imperial College London, London, UK; f Vascular Institute, St George's University Hospitals NHS Foundation Trust, London, UK; g Department of General, Visceral and Transplant Surgery, Eberhard-Karls-University Hospital Tuebingen, Tuebingen, Germany; h Department of Bariatric and Metabolic Surgery, Imperial College London, London, UK; i Department of Emergency Medicine, Inselspital, University Hospital of Bern, Bern, Switzerland

**Keywords:** COVID-19, Intensive care, ICU care, Citations, SARS-CoV-2

## Abstract

**Background:**

Since December 2019, the severe acute respiratory syndrome coronavirus 2 (SARS-CoV-2),causative pathogen of coronavirus disease 2019 (COVID-19), has triggered a pandemic with challenges for health care systems around the world. Researchers have studied and published on the subject of SARS-CoV-2 and the disease extensively. What is the significance of articles published, shared and cited in the early stages of such a pandemic?

**Materials and methods:**

A systematic literature search in a time frame of 12 months and analysis rating using Principle Component Analysis (PCA) and Multiple Factor Analysis (MFA) were performed.

**Results:**

The 100 most cited COVID-19 articles were identified. The majority of these articles were from China (n = 54), followed by United States of America (USA) (n = 21) and United Kingdom (UK) (n = 8). All articles were published in high-ranked, peer-reviewed journals, with research focusing onthe the diagnosis, transmission and therapy of COVID-19. The level of evidence of the 100 most cited COVID-19 articles on average was low.

**Conclusion:**

In the early stages of a pandemic, new and innovative research can emerge and be highly cited, regardless of the level of evidence.

## 1 Introduction

The onset of the COVID-19 pandemic was not only a test of resilience for the human race, but it also put scientists through their paces. In being a novel virus there was initally a lack of literature to aid the medical workforce; it fast became a race for scientists to contribute to the evidence-base to guide management of unwell patients accordingly. Newly proposed treatments based on anecdotal evidence were being used across the world, however policy-makers and those treating patients on the ‘front-line’ were unable to rely on such data alone for assurance that these novel treatments would be best for patient care. Some countries such as the UK with NICE guidelines, heavily rely on validated and peer-reviewed evidence in order to formulate treatment guidelines and regimens.

One of the largest barriers to clinical confidence in hastily published ‘COVID-19’ articles is the distinct lack of high hierarchical levels of evidence. Whilst this could largely be due to the lack of time alongside intense pressure to publish research, there may also be a general lack of understanding that results from case-studies of small sample sizes cannot be extrapolated to be true of entire populations.

This paper aims to highlight, understand and assess the top 100 most-cited articles published under the topic of COVID-19 through a systematic search using stringent inclusion and exclusion criteria. As shown in the results section, most papers originated from China (n = 54) and USA (n = 21). Difficulties with translations of Chinese papers were found to be an issue (although most were published in English), with their focus on diagnosis, mechanism, transmission and treatment, whilst Western papers focused only on transmission and treatment.

Using Principle Component Analysis (PCA) and Multiple Factor Analysis (MFA) of the filtered search results, this systematic review explores the possible correlations between objective metrics including: number of citations, density, article age, hierarchical evidence level and impact factors. Our findings suggest that pioneering evidence was published and subsequently heavily cited regardless of the level of evidence (mainly levels IV & V). We hope that this review will be of use to those contributing to the evidence base in future time-pressured scenarios such as subsequentnovel pathogen emergences.

## 2 Materials and methods

The Web of Science and the iSearch COVID-19 portfolio were utilised as effective tools for retrieval of citation information of published Covid-19 articles.

• The Web of Science provides comprehensive citation data for articles published in Medline, Web of Science Core Collection, BIOSIS Citation Index, KCI-Korean Journal Database, Russian Science Citation Index, and SciELo Citation index [[1], [2], [3]]. Topic fields of articles (title, abstract, author's keywords and keywords within a record) were searched for the following keywords:

“Wuhan Coronavirus” OR “Wuhan Seafood Market Pneumonia Virus” OR “COVID19” OR “COVID-19” OR “COVID-2019” OR “Coronavirus Disease 2019” OR “SARS-CoV-2” OR SARS2 OR “2019-nCoV” OR “2019 Novel Coronavirus” OR “Severe Acute Respiratory Syndrome Coronavirus 2” OR “2019 Novel Coronavirus Infection” OR “Coronavirus Disease 2019” OR “Coronavirus Disease-19” OR “Novel Coronavirus” OR “Coronavirus” OR “SARS-CoV-2019” OR “SARS-CoV-19”.

• The iSearch COVID-19 portfolio is the National Institute of Health's comprehensive source for publications related to COVID-19. It demonstrates cutting-edge analytical capabilities and is updated daily.

**Fig. 1:**
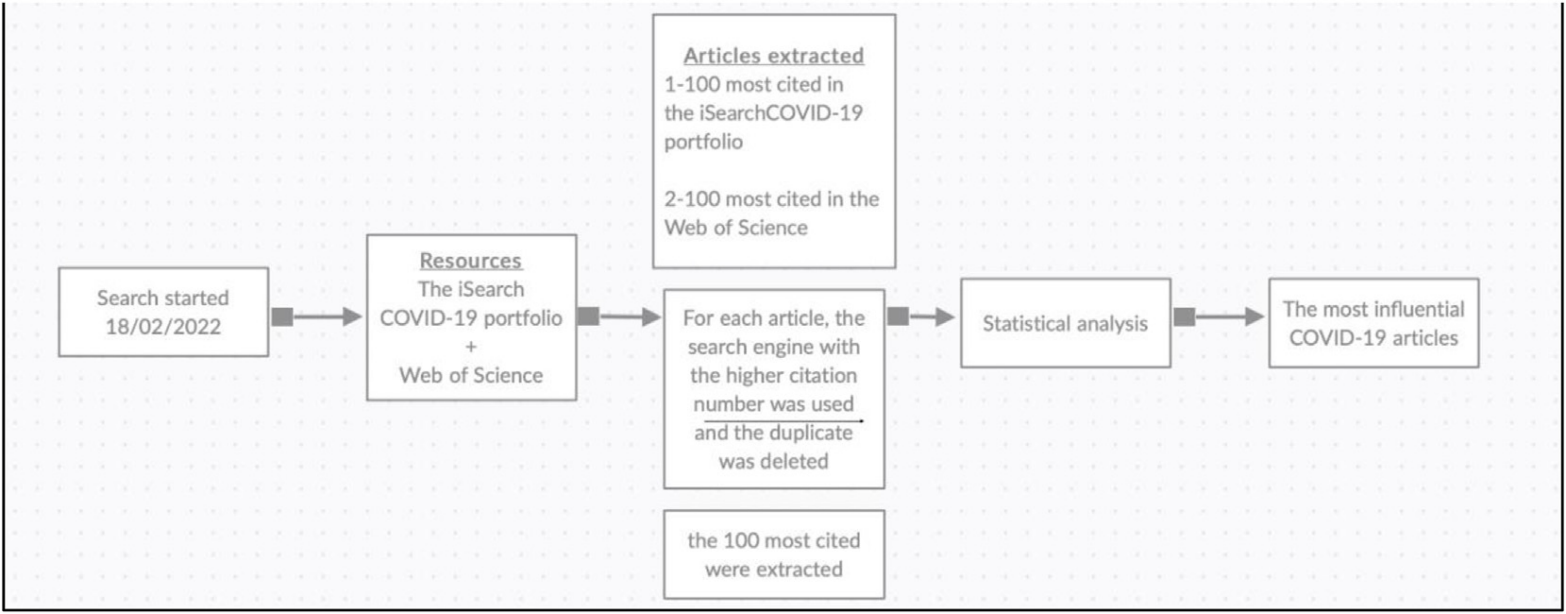
Flow diagram demonstrating the methodology and data extraction.

Only COVID-related articles submitted after 31/12/2019 (first reported COVID-19 case) were included in the study and the 100 most cited articles were identified and evaluated by two independent reviewers (Fig. 1).

COVID-19 articles were classified and assigned a level of evidence.

The levels of evidence (I–V) were adapted from the National Health and Medical Research Council (NHMRC) and The Centre for Evidence-Based Medicine (CEBM) [4].

Articles were categorized, using LitCovid, by different research topics as following:

Clinical Features, Mechanism, Diagnosis, Treatment, Transmission, Prevention, Forecasting and General [5].

### 2.1 Statistical analysis

Statistical analyses were conducted using the R programming language. Normality of data was checked using the Shapiro-Wilk test. The distribution of a parameter was characterised by the median and interquartile range. The Kendall rank correlation coefficient was used to measure the ordinal association between two values. Multiple Factor Analysis (MFA) was used to analyse quantitative variables simultaneously. A *P*-value of <0.05 was considered statistically significant. Microsoft Excel software was used for descriptive statistical analysis.

∗Any disagreements between the reviewers were resolved via consensus.

## 3 Results

Table 1 gives an overview of the top 100 most cited articles on COVID-19. All articles were published in 2020 (100%). The highest number of citations was 18958 and the lowest number was 1410. The median age of the articles was 21 months (range 13–24). In terms of levels of evidence - 14 articles were evidence level I, 7 were level II, 12 were III, 45 were IV and 22 were V.

**Table 1:** Overview of the top 100 cited COVID-19 articles (∗ next to rank number indicates systematic review).

Rank	Title	Citations(Density Citations/Age In months))	First Author	Last Author
1	Clinical features of patients infected with 2019 novel coronavirus in Wuhan, China.	18958(790)	Chaolin Huang	Bin Cao
2	Clinical Characteristics of Coronavirus Disease 2019 in China.	13699(652)	Wei-jie Guan	Nan-Shan Zhong
3	Clinical Characteristics of Covid-19 in China	13699(652)	Alexandre P. Zavascki	Diego R. Falci
4	Clinical course and risk factors for mortality of adult inpatients with COVID-19 in Wuhan, China: a retrospective cohort study.	12819(537)	Fei Zhou	Bin Cao
5	A Novel Coronavirus from Patients with Pneumonia in China, 2019	12182(530)	Na Zhu	Wenjie Tan
6	Clinical Characteristics of 138 Hospitalized Patients With 2019 Novel Coronavirus-Infected Pneumonia in Wuhan, China.	10228(445)	Dawei Wang	Zhiyong Peng
7	Epidemiological and clinical characteristics of 99 cases of 2019 novel coronavirus pneumonia in Wuhan, China: a descriptive study	10013(417)	Nanshan Chen	Li Zhang
8	A pneumonia outbreak associated with a new coronavirus of probable bat origin	9991(434)	Peng Zhou	Zheng-Li Shi
9	Characteristics of and Important Lessons From the Coronavirus Disease 2019 (COVID-19) Outbreak in China: Summary of a Report of 72,314 Cases From the Chinese Center for Disease Control and Prevention.	7855(457)	Zunyou Wu	Jennifer M. McGoogan
10	SARS-CoV-2 Cell Entry Depends on ACE2 and TMPRSS2 and Is Blocked by a Clinically Proven Protease Inhibitor.	7797(354)	Markus Hoffmann	Hannah Kleine-Weber
11	Early Transmission Dynamics in Wuhan, China, of Novel Coronavirus-Infected Pneumonia.	6343(288)	Qun Li	Zijian Feng
12	Genomic characterisation and epidemiology of 2019 novel coronavirus: implications for virus origins and receptor binding	5779(251)	Roujian Lu	Wenjie Tan
13	Antibody Responses to SARS-CoV-2 in Patients With Novel Coronavirus Disease 2019	5122(320)	Juanjuan Zhao	Zheng Zheng
14	Clinical course and outcomes of critically ill patients with SARS-CoV-2 pneumonia in Wuhan, China: a single-centered, retrospective, observational study.	4811(229)	Xiaobo Yang	You Shang
15	A familial cluster of pneumonia associated with the 2019 novel coronavirus indicating person-to-person transmission: a study of a family cluster	4651(194)	Jasper Fuk-Woo Chan	Kwok-Yung Yuen
∗16	The psychological impact of quarantine and how to reduce it: rapid review of the evidence	4599(200)	Samantha K Brooks	Gideon James Rubin
17	Presumed Asymptomatic Carrier Transmission of COVID-19	4555(207)	Yan, Bai	Meiyun Wang
18	Pathological findings of COVID-19 associated with acute respiratory distress syndrome	4519(205)	Zhe Xu	Fu-Sheng Wang
19	COVID-19: consider cytokine storm syndromes and immunosuppression.	4386(199)	Puja Mehta	Jessica J Manson
20	Aerosol and Surface Stability of SARS-CoV-2 as Compared with SARS-CoV-1	4362(198)	Neeltje Van Doremalen	Vincent J Munster
21	A new coronavirus associated with human respiratory disease in China.	4309(187)	Fan Wu	Yong-Zhen Zhang
22	Presenting Characteristics, Comorbidities, and Outcomes Among 5700 Patients Hospitalized With COVID-19 in the New York City Area.	4118(206)	Safiya Richardson	Karina W. Davidson
23	SARS-CoV-2 Viral Load in Upper Respiratory Specimens of Infected Patients	4079(185)	Lirong Zou	Jie Wu
24	Cryo-EM structure of the 2019-nCoV spike in the prefusion conformation.	3912(170)	Daniel Wrapp	Jason S. McLellan
25	Structure, Function, and Antigenicity of the SARS-CoV-2 Spike Glycoprotein	3750(170)	Alexadra C. Walls	David Veesler
26	Dexamethasone in Hospitalized Patients with Covid-19	3746(163)	Peter Horby	Martin J. Landray
27	Abnormal coagulation parameters are associated with poor prognosis in patients with novel coronaviruspneumonia	3737(170)	Ning Tang	Ziyong Sun
28	Risk Factors Associated With Acute Respiratory Distress Syndrome and Death in Patients With Coronavirus Disease 2019 Pneumonia in Wuhan, China.	3705(195)	Chaomin Wu	Yuanlin Song
29	The species Severe acute respiratory syndrome-related coronavirus: classifying 2019-nCoV and naming it SARS-CoV-2	3560(162)	Alexander E. Gorbalenya	John Ziebuhr
30	Remdesivir and chloroquine effectively inhibit the recently emerged novel coronavirus (2019-nCoV) in vitro	3473(151)	Manli Wang	Gengfu Xiao
31	Neurologic Manifestations of Hospitalized Patients With Coronavirus Disease 2019 in Wuhan, China	3467(173)	Ling Mao	Bo Hu
32	An interactive web-based dashboard to track COVID-19 in real time	3460(173)	Ensheng Dong	Lauren Gardner
33	Safety and Efficacy of the BNT162b2 mRNA Covid-19 Vaccine.	3413(263)	Fernando P. Plack	William C Gruber
34	Detection of 2019 novel coronavirus (2019-nCoV) by real-time RT-PCR	3205(134)	Victor M. Corman	Christian Drosten
∗35	A novel coronavirus outbreak of global health concern	3190(133)	Chen Wang	George F. Gao
36	Virological assessment of hospitalized patients with COVID-2019.	3100(155)	Roman Woelfel	Camilla Rothe
37	Immediate Psychological Responses and Associated Factors during the Initial Stage of the 2019 Coronavirus Disease (COVID-19) Epidemic among the General Population in China.	2829(123)	Cuiyan Wang	Roger C. Ho
38	A Trial of Lopinavir-Ritonavir in Adults Hospitalized with Severe Covid-19	2894(134)	Bin Cao	Chen Wang
39	First Case of 2019 Novel Coronavirus in the United States.	2787(121)	Michelle, Holshue	Satish K Pillai
40	Remdesivir for the Treatment of Covid-19 - Final Report.	2768(185)	John H. Beigel	H. Clifford Lane
41	Detection of SARS-CoV-2 in Different Types of Clinical Specimens.	2732(130)	Wenling Wang	Wenjie Tan
42	Correlation of Chest CT and RT-PCR Testing for Coronavirus Disease 2019 (COVID-19) in China: A Report of 1014 Cases	2721(146)	Tao Ai	Xia Liming
43	Hydroxychloroquine and azithromycin as a treatment of COVID-19: results of an open-label non-randomized clinical trial	2718(143)	Philippe Gautret	Didier Raoult
44	Endothelial cell infection and endotheliitis in COVID-19.	2718(129)	Zsuzsanna Varga	Holger Moch
45	Incidence of thrombotic complications in critically ill ICU patients with COVID-19	2683(141)	Frederikus A. Klok	Henrick Endeman
46	Factors Associated With Mental Health Outcomes Among Health Care Workers Exposed to CoronavirusDisease 2019	2550(116)	Jianbo Lai	Shaohua Hu
47	Baseline Characteristics and Outcomes of 1591 Patients Infected With SARS-CoV-2 Admitted to ICUs of the Lombardy Region, Italy.	2524(120)	Giacomo Grasselli	Antonio Pesenti
∗48	Coronavirus Disease 2019-COVID-19	2508(157)	Kuldeep Dhama	Alfonso J. Rodriguez-Morales
49	Clinical predictors of mortality due to COVID-19 based on an analysis of data of 150 patients from Wuhan, China	2491(119)	Qiurong Ruan	Song JX
50	The Incubation Period of Coronavirus Disease 2019 (COVID-19) From Publicly Reported Confirmed Cases: Estimation and Application	2442(116)	Stephen A. Lauer	Justin Lessler
51	The Epidemiological Characteristics of an Outbreak of 2019 Novel Coronavirus Diseases (COVID-19) - China, 2020	2427(106)	Zijian Feng	Jennifer M. McGoogan
∗52	Severe acute respiratory syndrome coronavirus 2 (SARS-CoV-2) and coronavirus disease-2019 (COVID-19): The epidemic and the challenges	2396(104)	Chih-Cheng Lai	Po-Ren Hsueh
53	Dysregulation of Immune Response in Patients With Coronavirus 2019 (COVID-19) in Wuhan, China	2366(131)	Chuan Qin	Dai-Shi Tian
54	Cancer patients in SARS-CoV-2 infection: a nationwide analysis in China.	2263(98)	Wenhua Liang	Jianxing He
55	Efficacy and Safety of the mRNA-1273 SARS-CoV-2 Vaccine.	2225(93)	Lindsey R. Baden	Tal Zaks
56	Comorbidity and its impact on 1590 patients with COVID-19 in China: a nationwide analysis	2223(106)	Wei-jie Guan	Jian-Xing He
57	Structural basis for the recognition of SARS-CoV-2 by full-length human ACE2.	2221(101)	Renhong Yan	Qiang Zhou
58	Pulmonary Vascular Endothelialitis, Thrombosis, and Angiogenesis in Covid-19.	2182(128)	Maximilian Ackermann	Danny Jonigk
59	Transmission of 2019-nCoV Infection from an Asymptomatic Contact in Germany	2133(93)	Camilla Rothe	Michael Hoelscher
60	Case-Fatality Rate and Characteristics of Patients Dying in Relation to COVID-19 in Italy	2083(99)	Graziano Onder	Silvio Brusaferro
∗61	The proximal origin of SARS-CoV-2	2068(94)	Kristian G. Andersen	Robert F. Garry
∗62	The epidemiology and pathogenesis of coronavirus disease (COVID-19) outbreak	2062(98)	Hussin A. Rothan	Siddappa N. Byrareddy
63	Clinical and immunological features of severe and moderate coronavirus disease 2019.	2057(98)	Guang Chen	Qin Ning
64	Structure of the SARS-CoV-2 spike receptor-binding domain bound to the ACE2 receptor.	2056(98)	jun Lan	Xinquan Wang
65	Receptor Recognition by the Novel Coronavirus from Wuhan: an Analysis Based on Decade-Long Structural Studies of SARS Coronavirus.	2048(93)	Yushun Wan	Fang Li
66	Nowcasting and forecasting the potential domestic and international spread of the 2019-nCoV outbreak originating in Wuhan, China: a modelling study	2003(87)	Joseph T. Wu	Gabriel M. Leung
67	Association of Cardiac Injury With Mortality in Hospitalized Patients With COVID-19 in Wuhan, China.	2000(105)	Shaobo Shi	Congxin Huang
68	Epidemiology of COVID-19 Among Children in China	1996(100)	Yuanyuan Dong	Shilu Tong
∗69	World Health Organization declares global emergency: A review of the 2019 novel coronavirus (COVID-19)	1972(90)	Catrin Sohrabi	Riaz
70	Clinical characteristics of 113 deceased patients with coronavirus disease 2019: retrospective study.	1972(90)	Tao Chen	Qin Ning
71	Critical Care Utilization for the COVID-19 Outbreak in Lombardy, Italy Early Experience and Forecast During an Emergency Response	1959(93)	Giacomo Grasselli	Maurizio Cecconi
72	Multidisciplinary research priorities for the COVID-19 pandemic: a call for action for mental health science	1948(97)	Emily A. Holmes	Ed Bullmore
∗73	The origin, transmission and clinical therapies on coronavirus disease 2019 (COVID-19) outbreak - an update on the status	1927(84)	Yan-Rong Guo	Yan Yan
74	Cardiovascular Implications of Fatal Outcomes of Patients With Coronavirus Disease 2019 (COVID-19)	1924(101)	Tao Guo	Zhibing Lu
75	Clinical characteristics and intrauterine vertical transmission potential of COVID-19 infection in nine pregnant women: a retrospective review of medical records	1904(83)	Huijun Chen	Yuanzhen Zhang
76	Factors associated with COVID-19-related death using OpenSAFELY	1886(105)	Elizabeth J. Williamson	Ben Goldacre
77	Temporal dynamics in viral shedding and transmissibility of COVID-19.	1879(89)	Xi He	Gabriel M. Leung
78	Anticoagulant treatment is associated with decreased mortality in severe coronavirus disease 2019 patients with coagulopathy.	1837(87)	Ning Tang	Ziyong Sun
∗79	The socio-economic implications of the coronavirus pandemic (COVID-19): A review	1738(87)	Maria Nicola	Riaz Agha
∗80	The trinity of COVID-19: immunity, inflammation and intervention	1719(86)	Mathew Zirui Tay	Lisa F.P. NG
81	Clinical characteristics of 140 patients infected with SARS-CoV-2 in Wuhan, China.	1718(90)	Jin-Jin Zhang	Ya-dong Gao
82	Temporal profiles of viral load in posterior oropharyngeal saliva samples and serum antibody responses during infection by SARS-CoV-2: an observational cohort study.	1715(82)	Kelvin Kai-Wang To	Kwok-Yung Yuen
83	Remdesivir in adults with severe COVID-19: a randomised, double-blind, placebo-controlled, multicentre trial.	1714(82)	yeming Wang	Chen Wang
84	Radiological findings from 81 patients with COVID-19 pneumonia in Wuhan, China: a descriptive study.	1660(76)	Heshui Shi	Chuansheng Zheng
85	Imbalanced Host Response to SARS-CoV-2 Drives Development of COVID-19.	1648(82)	Daniel Blanco-Melo	Benjamin R. tenoever
86	A SARS-CoV-2 protein interaction map reveals targets for drug repurposing.	1633(86)	David E. Gordon	Nevan J. Krogan
87	Characteristics and Outcomes of 21 Critically Ill Patients With COVID-19 in Washington State	1632(78)	Matt Arentz	Melissa Lee
88	Clinical management of severe acute respiratory infection (SARI) when COVID-19 disease is suspected. Interim guidance	1609(89)	Caroline Quach-Thanh	Titus Yeung
∗89	Prevalence of comorbidities and its effects in patients infected with SARS-CoV-2: a systematic review and meta-analysis.	1597(76)	Jing Yang	Yonging Zhou
90	A nationwide survey of psychological distress among Chinese people in the COVID-19 epidemic: implications and policy recommendations	1592(72)	Jianyin Qiu	Yifeng Xu
91	Tracking Changes in SARS-CoV-2 Spike: Evidence that D614G Increases Infectivity of the COVID-19 Virus.	1560(92)	Bette Korber	David C. Montefiori
∗92	COVID-19 and the cardiovascular system	1555(74)	Ying-Ying Zheng	Xiang Xie
∗93	The psychological impact of the COVID-19 epidemic on college students in China	1503(74)	Wenjun Cao	Jianzhong Zheng
∗94	WHO Declares COVID-19 a Pandemic.	1512(69)	Domenico Cucinotta	Maurizio Vanelli
95	Structural basis of receptor recognition by SARS-CoV-2.	1491(65)	Jian Shang	Fang Li
96	Estimates of the severity of coronavirus disease 2019: a model-based analysis.	1473(74)	Robert Verity	Neil M. Ferguson
97	Targets of T Cell Responses to SARS-CoV-2 Coronavirus in Humans with COVID-19 Disease and Unexposed Individuals.	1462(77)	Alba Grifoni	Alessandro Sette
98	Substantial undocumented infection facilitates the rapid dissemination of novel coronavirus (SARS-CoV-2).	1424(68)	Ruiyun Li	Jeffrey Shaman
99	Antibody responses to SARS-CoV-2 in patients with COVID-19.	1410(71)	Quan-Xin Lo	Ai-Long Hunag
100	Compassionate Use of Remdesivir for Patients with Severe Covid-19.	1405(70)	Jonathan Grein	Timothy Flanigan

**Fig. 2:**
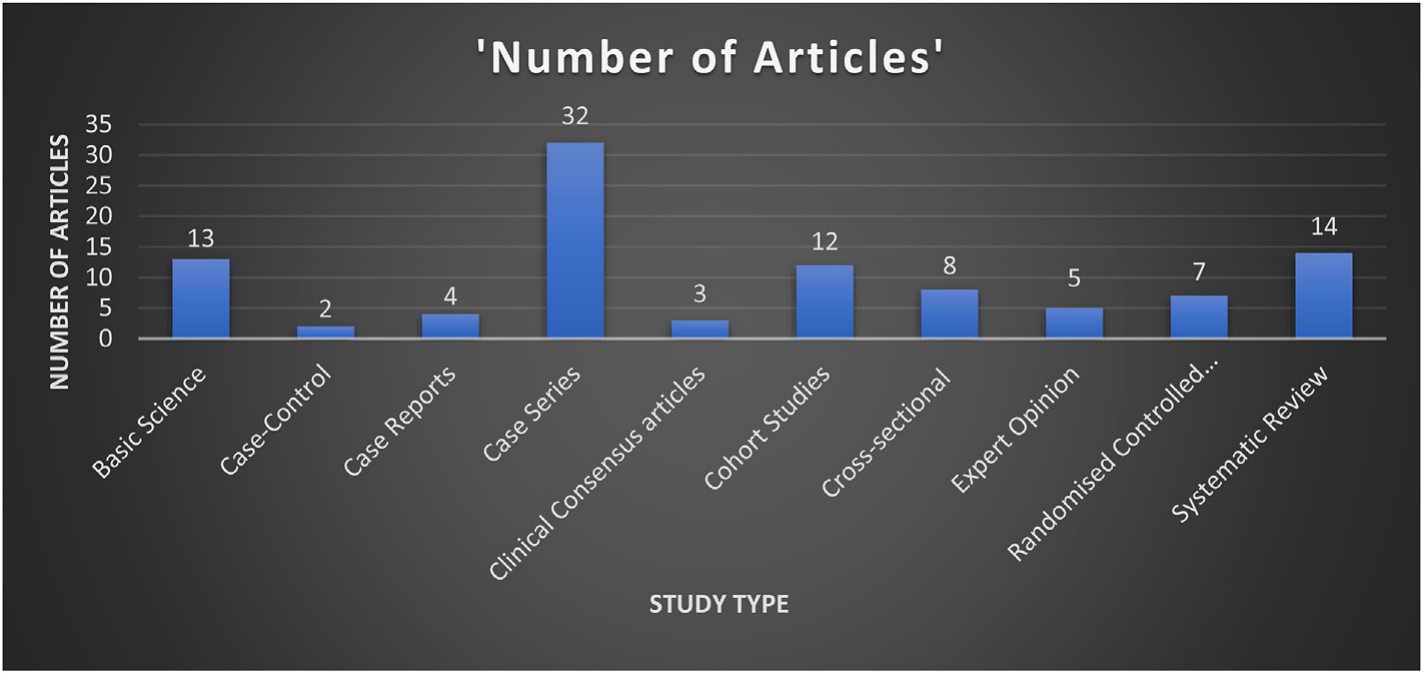
The hundred most cited Covid-19 articles study types.

13 articles were basic science, 2 case control studies, 4 case reports, 32 case series, 3 clinical consensus articles, 12 cohort studies, 8 cross sectional studies, 5 expert opinions, 7 randomised controlled trials and 14 systematic reviews (Fig. 2).

**Fig. 3:**
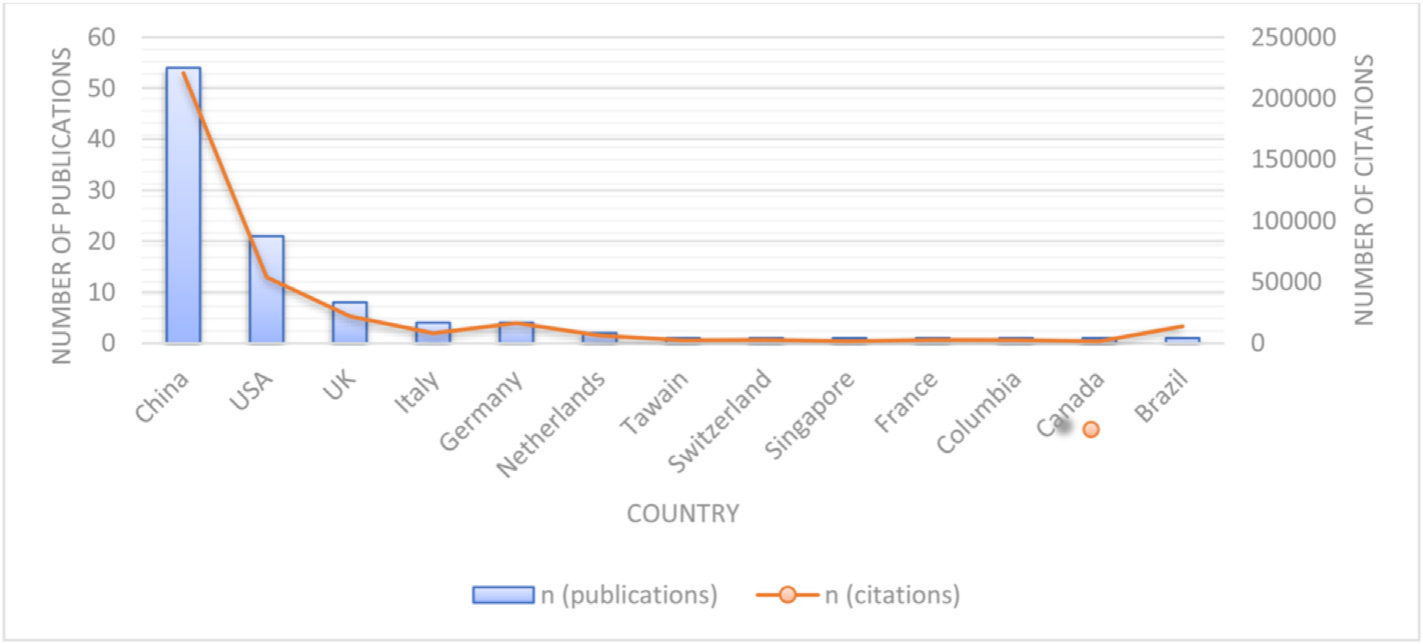
Number of publications and citations per country.

The Top 100 cited COVID-19 originated from 13 countries (Fig. 3), of which more than half were from China (n = 54), followed by United States of America (USA) (n = 21); United Kingdom (UK) (n = 8); Germany (n = 4); Italy (n = 4); Netherlands (n = 2); Brazil (n = 1); Canada (n = 1); Colombia (n = 1); France (n = 1); Singapore (n = 1); Switzerland (n = 1) and Taiwan (n = 1).

Table 2 shows the journals in which the top 100 cited COVID-19 articles were published with accompanying journal metrics. Of the 100 cited COVID-19 articles, 13 were published in the New England Journal of Medicine (NEJM), followed by 21 in the Lancet or associated journals (The Lancet (n = 13), Lancet Infectious Disease (n = 4), Lancet Respiratory medicine (n = 2), Lancet Psychiatry (n = 1), Lancet Oncology (n = 1)); 13 in Nature or associated journal (Nature (n = 7), Nature Medicine (n = 3), Nature Microbiology (n = 1), Nature Reviews Cardiology (n = 1), Nature Review Immunology (n = 1)); 13 in JAMA or associated journals (JAMA (n = 8), JAMA Neurology (n = 1), JAMA Network Open (n = 1), JAMA Cardiology (n = 2), JAMA Internal Medicine (n = 1)), 4 in Cell and 3 articles in Science.

**Table 2:** Journals in which top 100 cited COVID-19 articles were published with accompanying journal metrics.

Journals	Articles	Median Citation	Median Citation density	Country	Quartile (Impact factor)	Category Rank
Lancet Respiratory Medicine	2 Articles (2 level IV)	4665	217	UK	30.7	Q1
The Lancet	13 Articles (1 level I, 2 level II, 1 level III, 6 level IV, 3 level V)	4599	199	England	79.323	Q1
Clinical Infection Diseases	2 Articles (1 level III, 1 level IV)	3744	226	USA	9.079	Q1
JAMA Internal Medicine	1 Article (1 level III)	3705	195	USA	21.873	Q1
New England Journal of Medicine	16 articles (5 level II, 9 level IV, 2 Level V)	3580	185	USA	91.253	Q1
Nature Microbiology	1 Article (1 level V)	3560	162	UK	17.745	Q1
Cell Research	1 Article (1 level V)	3473	151	UK	25.617	Q1
JAMA Neurology	1 Article (1 level IV)	3467	173	USA	18.302	Q1
Eurosurveillance	1 Article (1 level V)	3205	134	Sweden	6.307	Q1
International Journal of Enviromental Research and Public Health	1 Article (1 level IV)	2829	123	Switzerland	3.39	Q1
Journal of Thrombosis and Haemostasis	2 Articles (1 level III, 1 level IV)	2787	129	UK	5.824	Q1
Radiology	1 Article (1 level IV)	2721	151	USA	11.105	Q1
Thrombosis Research	1 Article (1 level IV)	2683	141	USA	3.944	Q1
Journal of the American Medical Association	8 Articles (6 level IV, 2 level 5	2628	125	USA	56.274	Q1
International Journal of Antimicrobial Agents	2 Articles (1 level I, 1 level II)	2557	126	Netherlands	5.283	Q1
JAMA Network Open	1 Article (1 level IV)	2550	116	USA	8.485	Q1
Clinical Microbiology Reviews	1 Article (1 level 1)	2508	157	USA	26.132	Q1
Intensive Care Medicine	1 Article (1 level IV)	2491	119	USA	17.44	Q1
Annals of Internal Medicine	1 Article (1 level IV)	2442	116	USA	25.391	Q1
China CDC Weekly	1 Article (1 level IV)	2427	106	China	Na	Na
Lancet Oncology	1 Article (1 level III)	2263	98	England	41.316	Q1
The European Respiratory Journal	1 Article (1 level III)	2223	106	England	16.671	Q1
Science	3 Articles (1 level IV, 2 level V)	2221	101	USA	47.728	Q1
Journal of Autoimmunity	1 Article (1 level 1)	2062	98	UK	7.094	Q1
Journal of Clinical Investigation	1 Article (1 level IV)	2057	98	USA	14.808	Q1
Nature	7 Articles (1 level III, 3 level IV, 3 level V)	2056	105	UK	49.962	Q1
Journal of Virology	1 Article (1 level IV)	2048	93	USA	5.103	Q1
Paediatrics	1 Article (1 level IV)	1996	100	USA	7.125	Q1
British Medical Journal	1 Article (1 level IV)	1972	90	UK	14.093	Q1
JAMA Cardiology	2 Articles (1 level III, 1 level IV)	1962	103	USA	14.676	Q1
Lancet Psychiatry	1 Article (1 level V)	1948	97	UK	26.481	Q1
Military Medical Research	1 Article (1 level 1)	1927	84	UK	3.329	Q2
Nature Medicine	3 Articles (1 level I, 1 level III, 1 Level IV)	1879	89	USA	53.44	Q1
International Journal of Surgery	2 Articles (2 level II)	1855	88	UK	6.071	Q1
Nature Reviews Immunology	1 Article (1 level 1)	1719	86	UK	53.106	Q1
Allergy	1 Article (1 level IV)	1718	90	Denmark	13.146	Q1
Lancet Infectious Diseases	4 articles (2 level III, 1 level IV, 1 level V)	1689	79	USA	25.071	Q1
Pediatria i Medycyna Rodzinna	1 Article (1 level V)	1609	89	Poland	0.07	Q4
Cell	4 articles (1 level III, 3 level V)	1604	87	USA	41.584	Q1
International journal of Infectious Diseases	1 Article (1 level 1)	1597	76	Canada	3.623	Q2
General Psychiatry	1 Article (1 level IV)	1592	72	UK	2	Q3
Nature Reviews Cardiology	1 Article (1 level 1)	1555	74	UK	32.43	Q1
Psychiatry research	1 Article (1 level 1)	1553	74	UK	3.222	Q2
Acta Biomedica	1 Article (1 level 1)	1512	69	Italy	1.35	Q3

Articles published in China concentrated on the diagnosis, mechanism, transmission and treatment of COVID-19. On the other hand, articles published in Europe and the USA mainly focused on the transmission and the treatment of the virus (Table 3).

**Table 3:** Articles topic field.

Field	Country	Number of Articles
Clinical Features	Brazil	1
Diagnosis	China	6
Diagnosis	Germany	1
Diagnosis	Italy	1
Diagnosis	USA	1
Diagnosis AND Mechanism	China	7
Diagnosis AND Mechanism	USA	1
Diagnosis AND Prevention	China	1
Diagnosis AND Transmission	China	2
Diagnosis AND Transmission	USA	1
Diagnosis AND Treatment	China	2
Diagnosis AND Treatment	UK	1
Diagnosis AND Treatment	USA	1
Forecasting	China	1
Forecasting	Italy	1
Forecasting	UK	1
Forecasting AND Prevention AND Transmission	China	1
Forecasting AND Transmission	USA	1
General	China	6
General	UK	3
General	Italy	2
General	Netherlands	1
General	USA	1
Mechanism	China	8
Mechanism	USA	2
Mechanism	China	1
Mechanism	Switzerland	1
Mechanism AND Diagnosis	Germany	1
Mechanism AND Diagnosis AND Treatment AND Transmission AND Prevention	Canada	1
Mechanism AND Diagnosis AND Treatment AND Transmission AND Prevention	Columbia	1
Mechanism AND Diagnosis AND Treatment AND Transmission AND Prevention	UK	1
Mechanism AND Diagnosis AND Treatment AND Transmission AND Prevention	USA	1
Mechanism AND Prevention	China	1
Mechanism AND Transmission	China	2
Mechanism AND Treatment	USA	7
Mechanism AND Treatment	China	4
Mechanism AND Treatment	Germany	1
Mechanism AND Treatment	Singapore	1
Mechanism AND Treatment	UK	1
Mechanism AND Treatment AND Transmission	China	1
Transmission	China	2
Transmission	Germany	1
Transmission	USA	1
Transmission AND Prevention	China	1
Treatment	China	8
Treatment	USA	3
Treatment	France	1
Treatment	Netherlands	1
Treatment	UK	1
Treatment	USA	1
Treatment AND Transmission AND Prevention	Taiwan	1

**Fig. 4:**
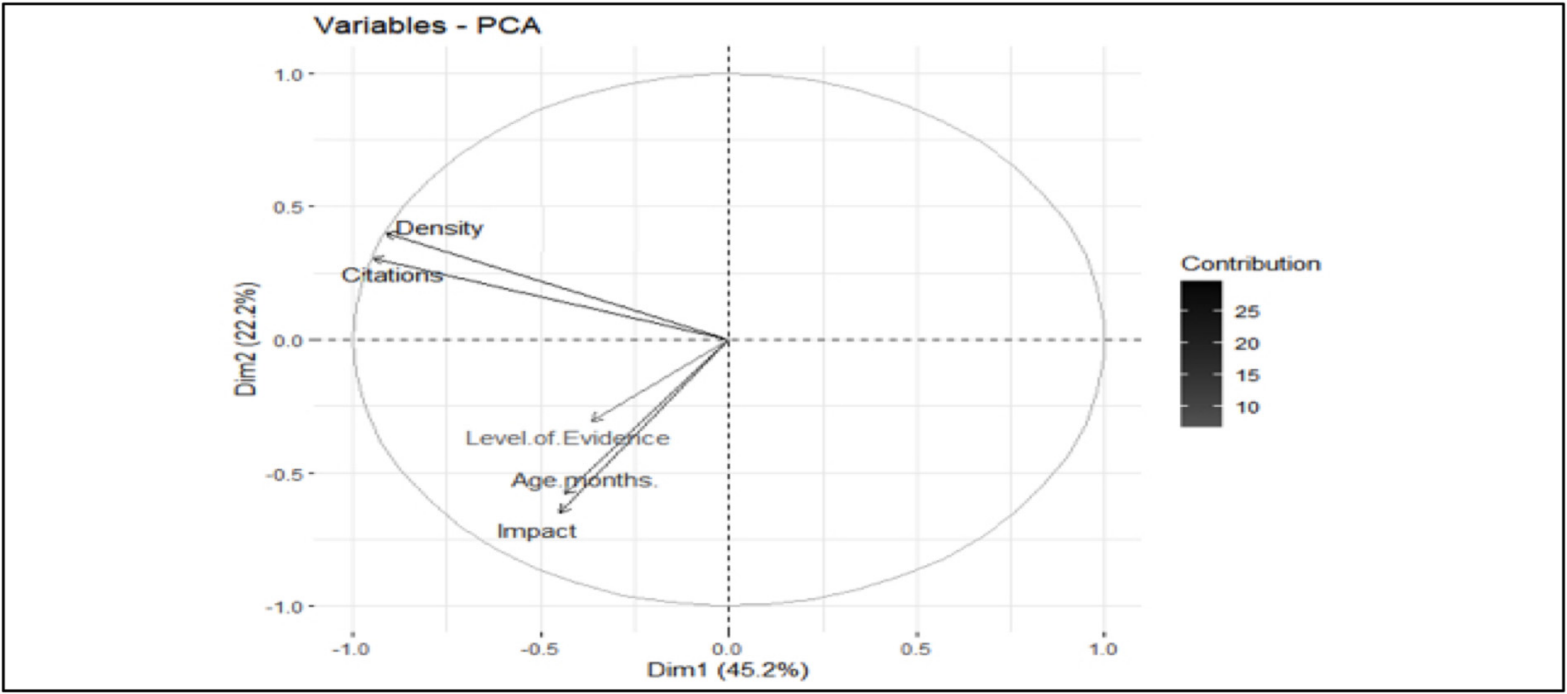
Principal component analysis of the relationship between citation number, citation density, level of evidence and Impact.

Principle Component Analysis (PCA) revealed a strong correlation between the number of citations and the citation density of citations. Furthermore, there was also a strong correlation between the age of the article, the level of the evidence and the impact factor. There was a significant trend towards increased frequency of citations with age of the article (r = 0.26, P = 0.0004). The number of citations an article had was not significantly associated with the level of evidence (r = 0.152, p = 0.152) (Fig. 4).

## 4 Discussion

This systematic review identified the 100 most cited articles on Covid-19 and sought to identify trends within them by applying citation analysis techniques. In late 2019, the COVID-19 pandemic presented one of the greatest challenges of the modern scientific era. With an estimated 503,862 deaths worldwide reported within the first 6 months of 2020 [6], the gravity and urgency of the problem required rapid advancement in knowledge to a degree not previously seen. It is unsurprising that with the amount of funding and resource invested, great volumes of scientific literature were produced in a relatively short period of time. What is surprising is the degree to which this occurred. Despite the first case of COVID-19 being only reported in December 2019, by July 2020 over 27,000 COVID-19 related articles had been published [7], with Covid related articles accounting for more scientific publication then all other topics combined. This unprecedented level of publication provokes many questions around the quality of research and the readiness of article acceptance [[8], [9], [10], [11], [12]].

All the articles were published in 2020 with a mean article age of 21 months (range 13–24 months). There was a weak but significant association between age and citation number; as citations take time to accumulate and consequently more recently published articles may not yet have achieved sufficient citations to have entered the review. The weakness of this relationship is likely a result of the short time-frame over which the articles have accrued the citations. The most highly cited article has been cited 18958 times and had a citation density of 790, the median citations was 2434.5 (IQR 1989.5–3749.0) and median citation density was 117.5 (IQR 89.5–185.2). This is particularly impressive as a variety of other citation classics have reported significantly lower median citations despite covering time periods of many years [2,[13], [14], [15], [16]]. On average a journal article will peak in citation density approximately 3 years after publication [17] which presents a potential problem in applying citation analysis to a novel and rapidly evolving field. The strong correlation between density and citation number combined suggests that highly cited articles continue to be cited and may be establishing ‘authority’ status. Given the ongoing expansion in literature there is a risk that articles, considered powerful by traditional metrics, may already be scientifically out of date but not yet past their peak in terms of citation accrual.

54% of the articles originated from China which is unusual for citation classics reviews. Similar reviews on other topics tend to draw most of their articles from the USA [1,2,[13], [14], [15]].This is likely explained by early geographic distribution of cases which would have granted a significant advantage for Chinese-based labs, resulting in earlier publication and thus citation accrual. Interestingly the USA provided almost half of the remaining articles, which allowing for the above explanation is in keeping with what would be expected. The early geographical distribution of cases may also explain diagnosis playing a significant role in articles from China but not from the rest of the world.

Articles representing level IV and V levels of evidence account for 67% of those identified. Whilst citation classics often demonstrate the inclusion of the lowest levels of evidence, it is seldom to this degree. For example, a review into general medical articles found 38% of articles were drawn from the lowest two levels [1] and another review into GI surgery 44% [2]. Only 7 RCTs were identified which is significantly lower than what would have been expected. It must be considered that higher levels of evidence such as RCTs (and systematic reviews of these) can take many months to conduct. It is probable that the lack of high-level evidence, and the over-representation of lower levels of evidence, is partially a result of the literature not yet reaching maturity. Another interesting finding of this review is the degree to which high impact factor journals are publishing low levels of evidence. It has been previously shown that in the top three general medical journals (The Lancet, New England Journal of Medicine and Journal of the American Medical Association) the level of evidence represented by an article regarding COVID-19 was significantly lower when compared to both contemporary and historic controls [9].

The main limitation of this review is the time at which it was conducted; this makes comparisons to similar reviews of different topics difficult. Due to the short publication span of the papers the definition of citation density had to be modified, using a reference period of a month rather than a year. It is likely that as the literature around COVID-19 matures trends in publications will change. It is possible that in the early stages of an emerging topic traditional citation metrics may not be the most reliable way of identifying the most influential research in the longer term. Presence on social media may play an important role in identification of future influential articles; number of tweets within the first 7 days of a publication are shown to correlate with high levels of citation [18]. The simple and easily repeatable methods of this review, however, allow for later comparative review to examine how these trends have changed.

## 5 Conclusion

This review has collated the 100 most influential COVID-19 papers and assessed trends within them. We have established that in the early phases of a pandemic new and ground-breaking research surfaces regardless of the evidence level and can gain high levels of citation.

## Funding

This study received funding from Insel Gruppe AG, Bern, Switzerland.

## CRediT authorship contribution statement

**Suhaib JS. Ahmad:** Conceptualization, Methodology, Literature search, Data curation, Project administration, Writing – original draft, Writing – review & editing, Validation, Final approval. **Konstantinos Degiannis:** Methodology, Writing – original draft, Writing – review & editing, Validation, Final approval. **Joseph Borucki:** Methodology, Writing – original draft, Writing – review & editing, Validation, Final approval. **Sjaak Pouwels:** Conceptualization, Methodology, Writing – original draft, Writing – review & editing, Validation, Final approval. **David Laith Rawaf:** Methodology, Writing – original draft, Writing – review & editing, Validation, Final approval. **Marion Head:** Methodology, Writing – original draft, Writing – review & editing, Validation, Final approval. **Chun Hei Li:** Methodology, Data curation, Visualization, Writing – original draft, Writing – review & editing, Validation, Final approval. **Rami Archid:** Methodology, Writing – original draft, Writing – review & editing, Validation, Final approval. **Ahmed R. Ahmed:** Methodology, Writing – original draft, Writing – review & editing, Validation, Final approval. **Anil Lala:** Methodology, Writing – original draft, Writing – review & editing, Validation, Final approval. **Wasif Raza:** Writing – original draft, Writing – review & editing, Validation, Final approval. **Katie Mellor:** Methodology, Writing – original draft, Writing – review & editing, Validation, Final approval. **Doerte Wichmann:** Writing – original draft, Writing – review & editing, Validation, Final approval. **Aristomenis Exadaktylos:** Conceptualization, Methodology, Supervision, Project administration, Funding acquisition, Writing – original draft, Writing – review & editing, Validation, Final approval.

## Declaration of competing interest

The authors declared no conflict of interest.
